# Author Correction: Integrated analysis of canine soft tissue sarcomas identifies recurrent mutations in *TP53*, *KMT* genes and *PDGFB* fusions

**DOI:** 10.1038/s41598-024-74722-9

**Published:** 2024-10-07

**Authors:** Sunetra Das, Rupa Idate, Susan E. Lana, Daniel P. Regan, Dawn L. Duval

**Affiliations:** 1 Department of Clinical Sciences, College of Veterinary Medicine and Biomedical Sciences, Colorado State University, Fort Collins, CO 80523 USA; 2 Flint Animal Cancer Center, Colorado State University, Fort Collins, CO 80523 USA; 3 Department of Microbiology, Immunology and Pathology, Colorado State University, Fort Collins, CO 80523 USA; 4 University of Colorado Cancer Center, Anschutz Medical Campus, Aurora, CO 80045 USA

Correction to: *Scientific Reports*10.1038/s41598-023-37266-y, published online 27 June 2023

In the original version of this Article the authors omitted a reference, which is listed below as Reference 15.

Ulvé R. et al. Discovery of Human-Similar Gene Fusions in Canine Cancers. *Cancer Res*; **77** (21): 5721–5727. https://doi.org/10.1158/0008-5472.CAN-16-2691 (2017).

This reference is now cited in the Introduction section, along with the following text addition:

“Similarly, a *COL3A1*-*PDGFB* fusion has been identified in a canine DP-like sarcoma^15^.”

As a result of the additional reference, the subsequent references have been renumbered.

The original Article has been corrected.

